# Severe Acute Pancreatitis in Pregnancy

**DOI:** 10.1155/2015/239068

**Published:** 2015-01-05

**Authors:** Bahiyah Abdullah, Thanikasalam Kathiresan Pillai, Lim Huay Cheen, Ray Joshua Ryan

**Affiliations:** ^1^Discipline of Obstetrics and Gynaecology, Faculty of Medicine, MARA University of Technology (UiTM), Jalan Hospital, 47000 Sungai Buloh, Malaysia; ^2^Hospital Sungai Buloh, Jalan Hospital, 47000 Sungai Buloh, Malaysia

## Abstract

This is a case of a pregnant lady at 8 weeks of gestation, who presented with acute abdomen. She was initially diagnosed with ruptured ectopic pregnancy and ruptured corpus luteal cyst as the differential diagnosis. However she then, was finally diagnosed as acute hemorrhagic pancreatitis with spontaneous complete miscarriage. This is followed by review of literature on this topic. Acute pancreatitis in pregnancy is not uncommon. The emphasis on high index of suspicion of acute pancreatitis in women who presented with acute abdomen in pregnancy is highlighted. Early diagnosis and good supportive care by multidisciplinary team are crucial to ensure good maternal and fetal outcomes.

## 1. Introduction

Acute pancreatitis in pregnancy is not an uncommon problem. The annual incidence of acute pancreatitis in general population is 5 to 80 per 100,000. However in pregnancy, it varies and is approximately 1 in 1000 to 1 in 10,000 [1–3]. More than 50% of cases in pregnancy are diagnosed in third trimester demonstrating that acute pancreatitis is more common with advancing gestational age, paralleling the frequency of gallstones in pregnancy [1].

Acute pancreatitis in pregnancy, as the name suggests, presents as an acute abdomen and can have a lethal effect on both the mother and the fetus. The high perinatal mortality and maternal mortality due to this condition have come down greatly due to the widespread use of ultrasound, Magnetic Resonance Imaging (MRI), and endoscopy as well as laparoscopy with multidisciplinary involvement in managing the condition.

We are reporting a case of acute pancreatitis in the first trimester of pregnancy which we initially diagnosed as ruptured ectopic pregnancy with a differential diagnosis of ruptured corpus luteal cyst. The final diagnosis was acute hemorrhagic pancreatitis with missed miscarriage.

## 2. Case Report

A 25-year-old gravida 4 para 3 at 8-week gestation presented to the emergency department with sudden onset of generalised abdominal pain and vomiting. There was no per vaginal bleeding, hematemesis, diarrhea or constipation, syncopal attack, or fever. She did not have any medical illness except for gastritis which occurred intermittently but was treated effectively with antacids and H_2_ antagonists by her family doctor.

On admission, she was conscious and in pain. She was normotensive and afebrile. There was tachycardia with pulse rate of 118 bpm. She had mild pallor but no jaundice. There was tenderness at the lower half of the abdomen with rebound tenderness but no guarding. No other significant findings were noted on physical examination.

Ultrasound examination showed a doubtful, very small intrauterine gestational sac with no fetal echo or yolk sac. There was significant amount of free fluids in the pelvic cavity; however, no adnexal mass was seen. A provisional diagnosis of ruptured ectopic pregnancy was made with ruptured corpus luteal cyst as the differential diagnosis. An emergency laparotomy was done and, intraoperatively, there was about 500 mL serosanguinous fluid in the peritoneal cavity and presence of ruptured corpus luteal cyst without any active bleeding.

Postoperatively, she was initially stable. However there were persistent abdominal pain and tachycardia of 130–150 bpm four hours after the laparotomy. Further investigations were done to look for other causes of her illness. Hemoglobin was still within normal range, from 16.6 to 15.5 g/dL (reference range: 12–15 g/dL), white cell count was raised at 19.7 × 109/L (reference range: 4.0–11.0 × 109/L), platelet count was normal (205 × 109/L reference range: 110–450 × 109/L), and hematocrit was normal (39.2% reference range: 37–47%). However, serum amylase, urine diastase, and lactate dehydrogenase were all raised. Serum amylase was 1273 U/L (reference range: 25–125), urine diastase was 3054 U/L (reference range: 1–17), and lactate dehydrogenase was 827 U/L (reference range: 125–220 U/L). Corrected calcium was 2.16 mmol/L (reference range: 2.1–2.55 mmol/L), and random blood sugar was 12.4 mmol/L (6.7–11.1 mmol/L). Serum albumin was low at 24 g/L (reference range: 35–50 g/L); bilirubin was raised at 46.8 *μ*mol/L (reference range: 3.4–20.5 *μ*mol/L). Other parameters of liver function test were normal. Additional tests including lipid profile, D dimer, thyroid function test, renal profile, coagulation screening, and electrocardiogram (ECG) were normal.

**Figure 1:**
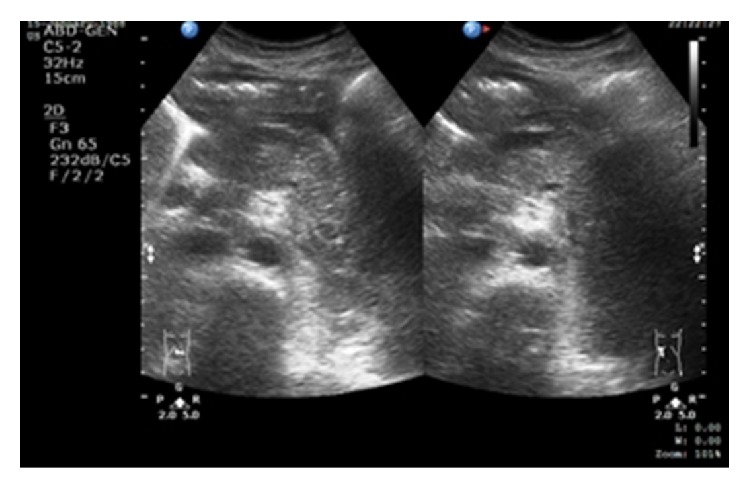
A bulky and inhomogeneous pancreas with presence of peripancreatic fluid in keeping with acute pancreatitis.

Ultrasound of the abdomen showed bulky pancreas with peripancreatic fluid suggestive of acute pancreatitis (see Figure 1). Therefore diagnosis of acute pancreatitis was made. Modified Glasgow Score was 3 at day 1 of admission.

Despite hydration and supportive management, tachycardia persisted and subsequently she developed Adult Respiratory Distress Syndrome (ARDS). Chest radiograph showed bilateral lower lobe haziness. She then required ventilation with CPAP and was nursed in ICU. Surgical team decided to perform a diagnostic laparoscopy to rule out any concomitant perforated gastric ulcer or perforated bowel. Intraoperatively, there was hemorrhagic fluid about 500 cc with saponification seen on the omentum with inflammation seen around the retroperitoneum region seen. The whole length of the bowel was normal. Peritoneal washout was done. Thus a diagnosis of acute hemorrhagic pancreatitis was made.

She was managed by a multidisciplinary team involving the intensivist, surgeon, gynaecologist, dietitian, and physiotherapist. She required assisted ventilation for five days. Her blood pressure remained stable without any inotrope. She was given intravenous morphine as painkiller. Reassessed after 48 hours later, the Modified Glasgow Score was 2. She also developed ileus, which required Ryle's tube and subsequently endoscopic nasoenteral tube (ENET) before it resolved. Postpyloric enteral feeding was commenced initially. Intravenous pantoprazole was also given. Intravenous Tazocin was given for 14 days.

Her general wellbeing and the blood test parameters improved remarkably. She was asymptomatic and the last blood test results prior to discharge were as follows: serum amylase dropped to 147 U/L (reference range: 25–125), serum LDH was 557 U/L (125–220 U/L), serum albumin was 37 g/L (35–50 g/L), and random blood sugar was normal.

She had complete miscarriage after a week of admission.

After 16 days staying in the hospital, she was well and was discharged home with further follow-up with the surgical team.

## 3. Discussion

Acute pancreatitis (AP) in pregnancy is most often associated with gallstone disease or hypertriglyceridemia [1, 4, 5]. Gallstones are the most common cause of acute pancreatitis during pregnancy, accounting for more than 70% of cases. Cholesterol secretion in the hepatic bile increases in the second and third trimester compared to bile acids and phospholipids leading to supersaturated bile. In addition, fasting and postprandial gallbladder volumes are greater with reduced rate of volume of emptying. This large residual volume of supersaturated bile in the gallbladder leads to cholesterol crystals and eventually gallstones [1]. Up to 10% of patients develop stones or sludge over the course of each pregnancy, obesity and increased leptin being risk factors [6]. Gall stones along with alcohol abuse account for more than 80% of cases of acute pancreatitis. Risk of acute pancreatitis from hypertriglyceridemia in pregnancy also seems to be the highest in third trimester and tends to be a more severe form of pancreatitis than that due to gallstones [7]. Pancreatitis in pregnancy may be associated with HELLP syndrome or preeclampsia leading to high fetal mortality or preterm delivery [8]. Other causes include drugs such as metformin [9] and sitagliptin [10]. Diabetes mellitus type 2 is associated with 2.8-fold higher risk [11]. Pregnancy itself can be a cause due to the physiological changes such as increasing weight, increased triglycerides, and increased levels of oestrogen. Hyperthyroidism, connective tissue diseases, infections, and trauma—both iatrogenic and accidental—are other rare causes of acute pancreatitis. However, primary diseases were absent in most cases (57.89%) [3]. Apart from being pregnant, this lady did not have other risk factors.

Acute pancreatitis in pregnancy is more difficult to diagnose in first trimester as compared to third trimester. The most common clinical presentations were abdominal pain (89.47%) and vomiting (68.42%) [3]. As the presentation of this patient was spontaneous acute onset of abdominal pain, vomiting, a short period of amenorrhoea, and presence of inconclusive findings of either a small empty intrauterine gestational sac or a pseudosac with free fluids on ultrasound, the diagnosis of ectopic pregnancy and differential diagnosis of ruptured corpus luteal cyst were not inappropriate. However, retrospectively, we believe acute pancreatitis was her primary problem since her initial presentation; however, as her clinical presentation mimicked other more common early pregnancy complications such as ruptured ectopic or ruptured corpus luteal cyst, therefore it was not thought as acute pancreatitis at the beginning of assessment. Interestingly, there was also a report on a patient of 7-week period of ammenorhea who was initially diagnosed to have acute pancreatitis, but later was found out to have an ectopic pregnancy [12]. It is important to highlight that preferably a diagnostic laparoscopy should have been performed rather than a laparotomy.

In evaluating pregnant patients with acute pancreatitis, it is suggested for four important questions to be answered, which are as follows. (1) Does this patient have acute pancreatitis (establishing the diagnosis and ruling out other causes)? (2) If it is acute pancreatitis what is the predicted severity (whether it is mild AP (MAP) or severe AP (SAP))? (3) Is there biliary aetiology? (4) What is the trimester of pregnancy? This last question will determine choice of imaging and mode of therapy [1].

Diagnosis of acute pancreatitis in this lady was established mainly by clinical presentation, blood markers, and ultrasound findings. The unresolved pain despite the initial intraoperative finding just showed a nonbleeding ruptured corpus luteal cyst which suggests there must be other causes of her abdominal pain. Serum amylase and/or lipase are useful blood marker in diagnosing acute pancreatitis. In this lady her serum amylase was very high which is adequate to establish the diagnosis, further being supported with the ultrasound findings later. Typically serum amylase concentration greater than three times normal is seen at presentation, which peaks in the first 24 h and falls to baseline in 3–5 days. In contrast, serum lipase concentrations are elevated for up to two weeks, making it a more sensitive and specific diagnostic test. However literature suggested that both enzyme concentrations were similar in nonpregnant and pregnant women and increase in either would be suggestive of acute pancreatitis in pregnancy [13, 14].

Mild acute pancreatitis (MAP), which is the most common form, has no organ failure or local or systemic complications and resolves in the first week. Severe acute pancreatitis (SAP) is defined by persistent organ failure, that is, organ failure for more than 48 hours. Local complications include peripancreatic fluid collection and peripancreatic or pancreatic necrosis [15].

We diagnosed the disease as severe acute pancreatitis (SAP) after the ultrasound clearly showed peripancreatic fluid and managed the patient in intensive care unit with the help of surgeons, intensivist, obstetricians, and the dietician. Ultrasound scan is safe and relatively inexpensive but it has low diagnostic value for acute pancreatitis. Another alternative imaging in cases of indeterminate ultrasound findings is magnetic resonance cholangiopancreatography (MRCP) without contrast medium which has over 90% sensitivity without exposing the mother and fetus to ionizing radiation. MRCP also limited the use of endoscopic retrograde cholangiopancreatography (ERCP) only to women who need therapeutic procedures. Endoscopic ultrasound has higher sensitivity than MRCP in visualization of choledocholithiasis and micro stones but it requires sedation [13, 14]. The repeat laparoscopy by the surgeon was also appropriate as it was to exclude other causes of acute abdomen. Before 1970s the diagnosis of acute pancreatitis in pregnancy was infrequent and vast majority of cases were diagnosed during surgery and/or autopsy [2, 16].

The initial management of acute pancreatitis in pregnancy does not vary from nonpregnant state. It consists of fluid restoration, oxygen, analgesics, and cessation of oral feeding to suppress exocrine function of pancreas, thereby preventing autodigestion of pancreas [17]. Conservative treatment is the preferred therapeutic method, in particular, for mild acute pancreatitis [4]. Management of acute pancreatitis due to gall stones and gallbladder disease in pregnancy does not vary from nonpregnant situations as well. Factors that may influence the management include the gestation of pregnancy, presence or absence of common bile duct dilatation, presence of cholangitis, and the severity of acute pancreatitis [1]. It has been recognised that cholecystectomy during second trimester is safe for mother and fetus. Indications for surgery in pregnancy are severe symptoms, obstructive jaundice, acute cholecystitis intractable to medical treatment, and peritonitis [1, 5]. Patients with hyperlipidemic pancreatitis should undergo lipid-lowering therapy, and hemofiltration should be done as soon as it becomes necessary [4].

In mild acute pancreatitis (MAP), nutritional support is not needed because the clinical course is usually uncomplicated and low fat diet can be started within 3–5 days [16]. In severe acute pancreatitis (SAP), treatments should include enteral feeding (EN) by either nasojejunal or postpyloric feeding and, if needed, they will require parenteral feeding. Total Parenteral Nutrition (TPN) feeding has a risk of infections and metabolic derangement, whereas enteral feeding (EN) is physiological and helps gut flora maintain gut immunity [17].

Though we have used antibiotics in this patient, prophylactic use of antibiotics in acute pancreatitis is controversial [2, 18]. There is no role for antibiotics in mild acute pancreatitis (MAP) but in severe acute pancreatitis (SAP) its role remains controversial. A systematic review and meta-analysis show antibiotic prophylaxis does not reduce mortality or protect against infected necrosis and frequency of surgical intervention [18].

Prognosis for mild acute pancreatitis (MAP) is excellent with no adverse effects on the fetus or mother. In 1973, maternal mortality due to acute pancreatitis in pregnancy was 31% [19] but in 2009 it came down to 1%. In the recent review of thirty-eight patients with acute pancreatitis, there were two reported maternal deaths [3] and, in another review of sixteen patients of this condition, there were two reported maternal deaths as well [4]. The perinatal mortality was 50% in 1973 but in a review in 2009 not even one perinatal death out of 73 patients with acute pancreatitis in pregnancy in second and third trimester and all 73 patients delivered term babies [16]. Despite that, the fetal risks from acute pancreatitis during pregnancy which include threatened preterm labour, prematurity, and in utero fetal death remain a concern [5]. Nevertheless, there are still obstetric problems to be addressed in the first trimester. Only 60% out of 30 patients with acute pancreatitis in first trimester achieved term pregnancy with fetal loss of 20% [16].

Management of severe acute pancreatitis (SAP) occurring in first trimester carries a better prognosis for mother but it is associated with increased fetal loss of about 20% [15]. In a study of 103 patients with acute pancreatitis in pregnancy, Banks et al. [15] found no maternal mortality in 30 patients in first trimester and only one maternal death in 96 patients studied. However the situation is not universal. Shoaib Gangat et al. [20] from Pakistan in their study of 166 patients with acute pancreatitis in pregnancy found 30.76% maternal mortality and 46% perinatal mortality.

The paradoxical trend in acute pancreatitis in pregnancy is the increase in the number of patients diagnosed but overall decline in perinatal and maternal morbidity and mortality associated with it. Increase in incidence can be attributed to various factors such as better diagnostic facilities, greater awareness of the disease, and increase in incidence of obesity all over the world. The advent of rapid assay methods for amylase, better supportive care of pancreatitis, newer therapeutic measures for gallstone pancreatitis, and overall improvement in antenatal care have definitely contributed to better maternal and fetal outcomes.
